# Viral whole-genome sequences of *Pseudomonas* jumbo phages, ΦNK1 and ΦBrmt, from sewage water in Japan

**DOI:** 10.1128/mra.01044-23

**Published:** 2025-06-20

**Authors:** Jumpei Fujiki, Tomohiro Nakamura, Natsuko Ichikawa, Kohana Tamamura, Haruka Yamamoto, Yoshiaki Sakata, Keisuke Nakamura, Hidetomo Iwano

**Affiliations:** 1 Laboratory of Veterinary Biochemistry, Rakuno Gakuen University School of Veterinary Medicine 13022, Ebetsu, Japan; 2 Phage Therapy Institute, Comprehensive Research Organization, Waseda University 593275, Tokyo, Japan; 3 Research Center for Drug and Vaccine Development, National Institute of Infectious Diseases 13511, Tokyo, Japan; 4 Laboratory of Small Animal Surgery, School of Veterinary Medicine, Azabu University 47710, Kanagawa, Japan; Queens College Department of Biology, Queens, New York, USA

**Keywords:** bacteriophage, phage therapy, jumbo phage, *P. aeruginosa*, phikzvirus

## Abstract

Jumbo phages have potentials for uncovering phage diversity. Here, we report the complete genome sequences of two *Pseudomonas* jumbo phages, ΦNK1 and ΦBrmt, isolated from waste water in Japan. To explore their molecular characteristics, whole phage genomes were sequenced and assembled via the short- and long-read platforms.

## ANNOUNCEMENT

Jumbo phages, with genomes ranging from 200 to 500 kbp, offer valuable insights into phage biology and diversity (1, 2). The first identified jumbo phage, *Pseudomonas* virus ΦKZ, has a 280,334 bp genome and a large capsid (145 nm) (3, 4). Despite their size and complexity, many aspects of jumbo phage gene functions remain unclear.

To further explore jumbo phages, we isolated two *Pseudomonas* phages, ΦNK1 and ΦBrmt, from Japanese wastewater collected in Niigata Prefecture and from a mixture of samples from Kochi and Hokkaido Prefectures, respectively, using the double-layer agar method (5). After centrifugation (10,000 × *g*, 15 min), sewage samples were syringe-filtered through a 0.45 µm filter (Advantec, Tokyo, Japan) and incubated with a *P. aeruginosa* Pa12 brawn mutant strain, previously isolated from a canine skin lesion and exhibiting resistance to LPS-targeting phages (6, 7). Subsequently, individual plaques were picked and purified through three rounds of plating. Isolated phages were amplified by the double-layer method and purified via a 100 kDa Amicon Ultra membrane filter (Merck, Darmstadt, Germany) based on the phage on tap (PoT) method (8). The purified phage samples underwent DNase treatment following the manufacturer’s instructions using the TURBO DNase free kit (Thermo Fisher Scientific, San Jose, CA, USA). The phage genomes were isolated using a phage DNA isolation kit (Norgen, Thorold, Canada).

**Fig 1:**
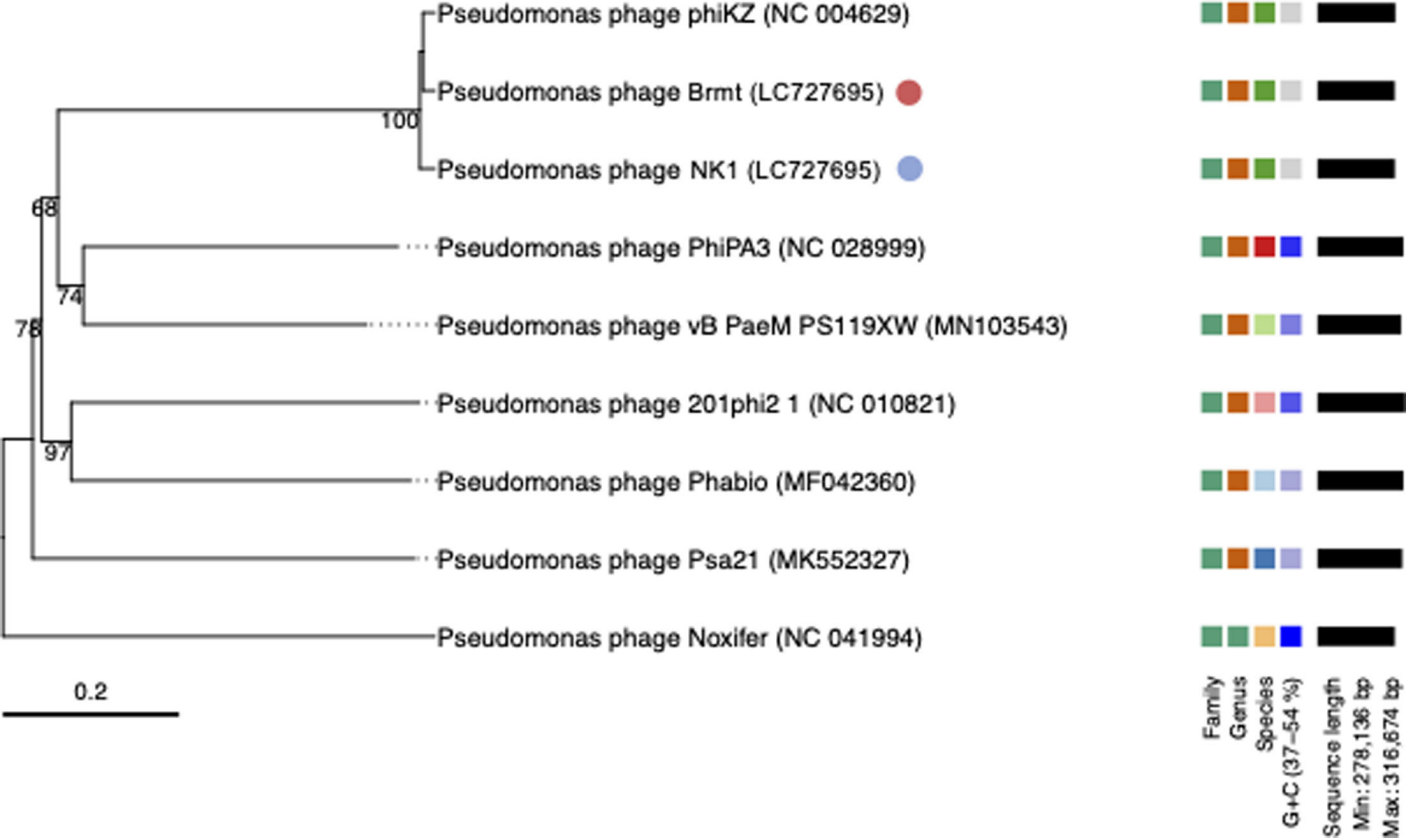
The phylogenetic tree was constructed and visualized by VICTOR (13) with default parameters. Whole-genome sequences of 7 *Pseudomonas* jumbo phages, representing master species classified by the International Committee on Taxonomy of Viruses (ICTV), were downloaded from the National Center for Biotechnology Information (NCBI). The blue circle indicates ΦNK1, and the red circle indicates ΦBrmt.

Libraries for short-read sequencing were prepared based on the manufacturer’s guidelines using a Nextera XT DNA Library Preparation kit (Illumina, San Diego, CA, USA). The whole genome was then 300 bp paired-end sequenced on the MiSeq platform, yielding 1,947,932 reads for ΦNK1 and 937,386 reads for ΦBrmt. To ensure data quality, FastQC v0.11.9 and trimmomatic v0.39 were used for adapter clipping, quality trimming (LEADING:20 TRAILING:20 SLIDINGWINDOW:4:15), and excluding reads below a minimum length of 50 bp (9). In addition, libraries for long-read sequencing were prepared using the SMRTbell Prep Kit 3.0 (PacBio, Menlo Park, CA). The resulting samples were sequenced on the PacBio Revio platform, yielding 141,392 HiFi reads for ΦNK1 and 82,119 HiFi reads for ΦBrmt. The trimmed sequence reads were *de novo* assembled by Unicycler v0.4.8 (10), resulting in a single contig for each phage. For ΦNK1, the assembly yielded a contig of 278,676 bp with a G + C content of 36.8%, an average long-read coverage of 4,271×, and an average short-read coverage of 483×. For ΦBrmt, the contig was 278,203 bp with a G + C content of 36.8%, an average long-read coverage of 2,344×, and an average short-read coverage of 420× (Table 1). Termini could not be predicted using PhageTerm (11); however, hybrid genome assembly using short- and long-read sequencing suggests circular permutation, as determined by Unicycler, indicating a linear genome organization similar to ΦKZ. Finally, the assembled contig was annotated using DFAST v1.1.0 (12). Default parameters were used for all software except where otherwise noted. Phylogenetic VICTOR analysis (13) showed that ΦNK1 and ΦBrmt cluster closely with the *Pseudomonas* jumbo phage ΦKZ, supporting their classification within the genus *Phikzvirus* (Table 1; Fig. 1).

**TABLE 1:** Characteristics of the annotated *Pseudomonas* viruses, *ΦNK1 and ΦBrmt*

Isolated phages	Length (bp)	G + C content (%)	No. of CDSs	No. of tRNA	Coding ratio (%)	Related master species	Genus
ΦNK1	278,676	36.8	360	7	91.7	ΦKZ	*Phikzvirus*
ΦBrmt	278,203	36.8	362	7	91.8	ΦKZ	*Phikzvirus*

## Data Availability

Complete genome sequences of ΦNK1 and ΦBrmt are available in DDBJ/ENA/GenBank under accession number LC727701.1 and LC727695.1. Illumina sequence reads were deposited in the Sequence Read Archive (SRA) under accession numbers DRR399903 and DRR399897; PacBio sequence reads under accession number DRR657713 and DRR657714, respectively.
